# Agriculture land-use change is driven by socioeconomic flows across local to global scales

**DOI:** 10.1016/j.isci.2026.115200

**Published:** 2026-03-02

**Authors:** Joris Van Zeghbroeck, Michele Remer, Nicholas Manning, Emilio F. Moran, Jianguo Liu

**Affiliations:** 1 Center for Systems Integration and Sustainability, Department of Fisheries and Wildlife, Michigan State University, East Lansing, MI 48823, USA

**Keywords:** Environmental science, Agricultural economics, Social sciences

## Abstract

Agriculture is the key to global food security and environmental sustainability; however, little is known about how cross-scale socioeconomic flows influence agricultural land use. We addressed this knowledge gap by evaluating the metacoupled flows of crops, capital investments, and migration. We assessed how these flows impacted land used for crop production in India and Argentina, which contain global biodiversity hotspots and high levels of agriculture. During our study time frame (1990–2015), land used for crop flows increased by 33.6 Mha, primarily driven by increases in crops produced for domestic consumption. Capital investments from distant countries and within the focal countries increased transportation infrastructure and processing capacity, supporting the expansion of crop production. Migration from adjacent countries and within the focal countries increased urban populations, leading to the displacement of agricultural lands surrounding metropolitan areas. Our results underscore the significance of cross-scale socioeconomic flows and their potential to inform effective land use policies.

## Highlights

• Intracoupled crop flows in India and Argentina drove changes in cropland area
• Cropland use was affected by crop, capital investment, and migration flows
• Distant and domestic capital investments supported crop production infrastructure
• Cross-scale migration increased urban land area, resulting in land displacement

## Introduction

Since the Industrial Revolution, human populations have converted approximately 3 billion hectares of natural landscapes into cropland, pasture, or urban areas through drastic increases in land-use change (LUC).^1^ LUC is a dynamic process in which a single parcel of land can be converted into multiple land use categories. An estimated 32% of the global land area has undergone at least one gross LUC between 1960 and 2019, which was four times higher than previous estimates.^2^ Agricultural production has been identified as one of the leading drivers of LUC and degradation, resulting in negative impacts on social, political, cultural, and economic systems.^3^ Increased production over the last 60 years has made agriculture the largest land-use category globally, occupying 46% of habitable land.^4^ Agricultural land use continues to expand as a growing global population and changing global diets (e.g., increased meat consumption) increase crop demand and the land required for crop production.^3^^,^^5^^,^^6^^,^^7^ Higher global demand for crops leads to agricultural expansion often taking place in species-rich ecosystems, causing biodiversity loss, higher levels of greenhouse gas emissions, changes to terrestrial water cycles, and soil carbon loss.^8^^,^^9^^,^^10^^,^^11^^,^^12^ As agricultural land use continues to expand, it is crucial to understand the processes driving LUC to minimize negative environmental impacts globally.

LUC is driven by an interconnection of complex processes ranging from local to global scales and affects coupled human and natural systems.^13^^,^^14^ LUC research has historically focused on single drivers at varying scales, often only evaluating them separately at either global (across distant global regions), regional (within a global region), or national or local (within a country) scales.^15^ To address the complexities present in LUC, recent studies have begun to incorporate coupled systems and nexus approaches, which are the integration of solutions across distinct areas (sectors, scientific fields, disciplines, industries, etc.) that focus on the connections, synergies, and tradeoffs of complex systems.^16^^,^^17^^,^^18^^,^^19^^,^^20^ For example, Alexander et al.^17^ evaluated how the diet-population-energy-yield nexus impacted LUC. Across this nexus, the researchers identified diet and energy as the leading drivers of higher land use for crop production, and concluded that shifting demand would be the key to managing future LUC. Past studies that have implemented nexus approaches have helped identify interactions across multiple factors and how they impact LUC.^16^ However, these approaches did not always account for spatial variability across scales.

**Figure 1:**
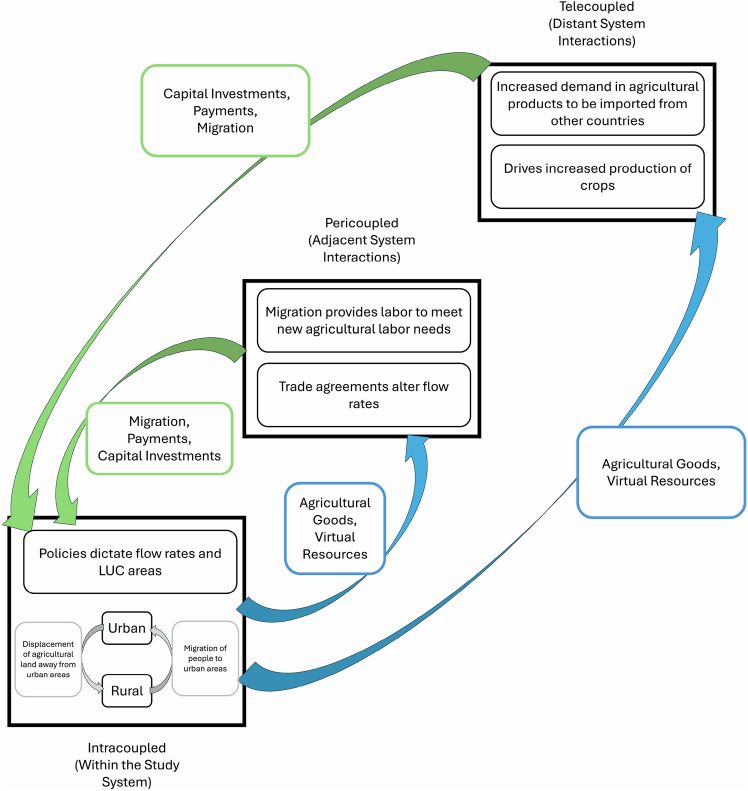
Conceptual diagram of metacoupled drivers behind LUC Arrows indicate flows between and within systems (such as countries, represented by rectangle boxes) with blue arrows representing the flow of agricultural goods and virtual resources from the focal system to distant and adjacent systems. The green arrows represent flows of migrants and capital from distant and adjacent systems to the focal system. Gray arrows indicate intracoupled flows (within the focal system).

The metacoupling framework can help identify LUC drivers across spatial scales, as it was previously used to support the evaluation of multiple socioeconomic factors’ effects on complex global sustainability issues.^21^ The framework helps researchers to evaluate cross-scale interactions between coupled human and natural systems (e.g., sending, receiving, and spillover systems) by assessing flows (e.g., the movement of energy, material, people, and/or capital) in conjunction with agents (entities involved with system interaction), causes (reasons for system interaction), and effects (consequences of system interaction)^22^ (Figure 1). Metacoupling consists of human-nature interactions within a system (intracoupling), between adjacent systems (pericoupling), and between distant systems (telecoupling) (Figure 1). The metacoupling framework can be used to evaluate the causes of flow shifts over time and the effects of these changes. Furthermore, by identifying the agents controlling the flows, actionable policies can be developed to alter the flows and their effects. By integrating these methods across couplings (intra-, peri-, and tele-coupling), researchers can gain insights into the cross-scale socioeconomic drivers of LUC.

Previous metacoupling research has focused on flows of materials and goods, ecosystem services, physical resources, and virtual resources, leading to the identification of new environmental and social impacts between systems.^23^^,^^24^^,^^25^^,^^26^ However, few studies have evaluated socioeconomic flows, including the movement of financial and human resources such as financial capital investment and labor migration, which are essential to agricultural production and have the potential to be key drivers of LUC.^21^ Evaluating these flows and their effects across scales is important for understanding LUC, as dynamics can differ across couplings with unique agents, causes, and effects (Table 1). Because metacoupling research is inherently complex, operationalizing the framework involves dividing our studies into smaller projects before they are eventually synthesized. Therefore, we present this research as a starting point for considering cross-scale flows, while future studies can examine other aspects of the framework to synthesize all components (Table 1).

**Table 1:** Cross-scale interactions

Focal system	Partner system	Flow	Cause	Agents	Effects
Study country	Country sending or receiving flow	Crops	The shifting demand of importing countries changes the amount of crops produced and the land required for crop production (T/P/I)	Consumers Producers Corporations	Increased land is used for crop flows due to increased demand and economic incentives for production.
		Migrations	Migration (typically from lower-income to higher-income countries) supports labor in agricultural production (P) Migration increases the total population, increasing crop demand (P/T) Migration from rural to urban areas leads to a shift in urban land requirements and crop flows (I)	Migrants Governments	Agricultural production is often dependent on migrant labor. Increased labor can either expand production (resulting in increased land use) or intensify production (maintaining or decreasing land use). Larger populations can require more crops, potentially leading to increased land use for crop production. Urban land expansion, due to migration, can lead to the development of agricultural lands displacing them to other parts of the country.
		Capital investments	Corporations and banks invest in land, infrastructure, and agrobusinesses based on economic opportunities (T/P/I)	Corporations Governments	Increased capital investments can directly expand crop production (through land grabbing), indirectly support cropland expansion (by investing in infrastructure and processing facilities), or reduce cropland expansion (by investing in improved agricultural systems).

Metacoupling of LUC based on three major categories of flows. The primary coupling types for the causes are indicated by T (telecoupling), P (pericoupling), and I (intracoupling).

Despite advances in understanding the drivers of LUC, a knowledge gap remains regarding how cross-scale socioeconomic flows influence LUC within an intracoupled focal country. To address this gap, our paper aims to demonstrate how the metacoupling framework can be utilized to evaluate LUC by assessing how capital investment (investments in land, infrastructure, processing facilities, or other crop production and distribution elements), migration (movement of people from one location to another), and crop (cultivated products used as food or raw materials) flows impacted LUC in Argentina and India between 1990 and 2015. These countries were selected since they have received disproportionately less research attention than their neighboring countries (Brazil and China), despite their significance in global food trade,^27^^,^^28^^,^^29^ international influence,^30^^,^^31^^,^^32^ and biodiversity resources.^33^^,^^34^ Additionally, both have significant global importance as India became the most populous country in the world in 2023,^35^ while Argentina has been a historic “super-exporter” of crops to the global market.^36^ These two countries also differ in significant ways, including crop diversity, agricultural regions, population size, climate, and trading partners, which can yield insights into the unique metacoupled dynamics of different systems. The specific objectives of this paper are to (1) outline a methodology for evaluating metacoupled socioeconomic flows and their influence on LUC; (2) identify the intracoupled, pericoupled, and telecoupled flows that influence LUC in the focal countries between 1990 and 2015; and (3) illustrate the cross-scale impacts of socioeconomic flows on LUC. Through this research, we present a conceptual integration of multiple factors affecting LUC, factors that have not been previously considered or are scattered across the literature. By integrating various data sources, we systematically report the land used for intracoupled, pericoupled, and telecoupled crop flows while identifying potential cross-scale drivers.

## Results

### Telecoupled drivers of land-use change

As agricultural systems become more globally integrated, telecouplings (interactions between distant systems) have repeatedly been identified as important causes of LUC.^37^ From 1961–2022, global agricultural LUC was predominantly driven by population growth and increases in the production of animal products, leading to expansions of rangelands and land devoted to crop production.^17^ Animal production has been attributed to 65% of total LUC and has been driven by globalization, higher global incomes, and diet changes.^17^ For example, Brazil’s accelerated soybean production was driven by higher affluence rates in China, as soybeans grown in Brazil provided animal feed for livestock consumed in China.^38^ Researchers have also studied how telecoupled crop flows negatively impacted biodiversity and caused nutrient pollution, specifically within agricultural sending systems, rather than within the receiving system.^17^^,^^39^^,^^40^^,^^41^^,^^42^ However, crop flows related to shifting global demands are not the only telecouplings that can impact LUC. Telecoupled capital investments^43^ and migration^44^^,^^45^^,^^46^ have received substantially less research than crop flows.

Telecoupled capital investments, including investments in agricultural production and infrastructure as well as direct land purchases, have affected land use in diverse regions, including the Amazonian region, the Congo basin, sub-Saharan Africa, and the east Asia/Pacific.^13^^,^^15^^,^^47^^,^^48^ In 2010, Brazil had 34,371 rural estates (totaling 4.35 Mha) owned by foreign investors, with 2.79 Mha owned by investors from distant countries.^49^ Capital investment flows are required to achieve food security goals, requiring an estimated $80 billion USD.^50^

Migration can also be considered as a telecoupling. The International Organization for Migration reported that 244 million people were international migrants in 2015.^51^ Migration can occur for many reasons (personal, financial, political, etc.), which can impact whether an individual is likely to migrate within their country (intracoupling), to an adjacent country (telecoupling), or to a distant country (telecoupling). One study found that less affluent migrants were more likely to migrate within their own country or to an adjacent country, compared to more affluent migrants, who tended to move to distant countries.^51^ These migration flows can have substantial impacts on agricultural systems, impacting LUC. For example, migration from Guatemala to the US led to a reduction in farmland and an increase in forested land in Guatemala.^52^ Each of the three highlighted telecoupled flows impacts LUC by affecting global markets, global demand, or infrastructure related to agricultural production (Table 1).

### Telecoupled impacts on land-use change in Argentina

**Figure 2:**
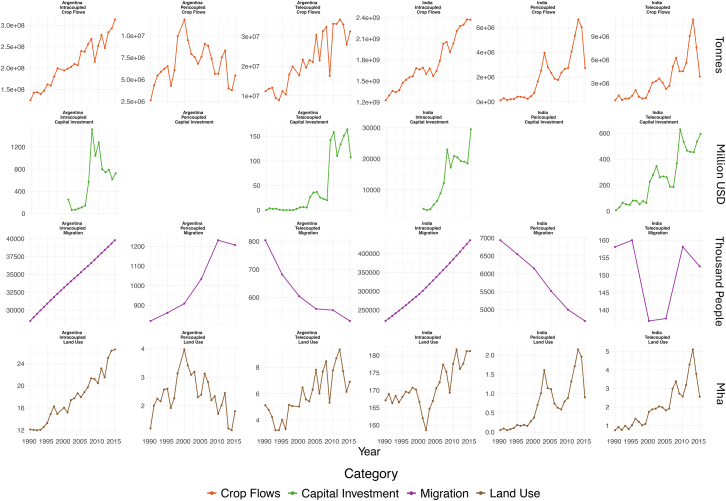
Change in flows over time The change in metacoupled flows over time, by coupling and country. Pericoupled capital investment flows were not included due to a lack of available data. Intracoupled migration is represented by urban population due to a lack of available data.

Telecoupled crop flows and capital investment have increased in Argentina, leading to higher rates of land use for telecoupled crop flows. In 2015, Argentina used 6.91 Mha/yr of cropland for telecoupled exports, sending 47 crops to 142 distant countries. This represented a rise of 1.8 Mha/yr, nine crops, and 60 countries compared to 1990. Land used for telecoupled crop flows showed a significant (*p* < 0.05) positive trend of 0.18 Mha/yr over time. In total, changes in land used for telecoupled crop flows accounted for 10.7% of the increase in land used for crop flows between 1990 and 2015 and 19.6% of the harvested crop land area in 2015 (Figure 2). Between 1990 and 2015, the majority of Argentina’s crop exports were sent to distant countries and regions, with Central and East Asia, as well as Europe, receiving food products and vegetables worth over $15 billion USD in 2015.^53^

The expansion of telecoupled crop exports can be partly attributed to demand from receiving countries. For example, demand for biofuel in the US, the European Union, and over 30 other countries was driven by policy mandates, leading to crops being used for fuel rather than food. Additional increases in global demand for animal-based proteins, particularly in China and Brazil, have also driven agricultural land conversion.^38^ To meet these demands, between 1990 and 2015, Argentina increased telecoupled crop flows of corn and soybeans by 14.2 and 8.7 Mtonnes per year, respectively, which was associated with an additional 3.9 Mha/yr of land area used for telecoupled crop flows. Across the same time period, several crops saw substantial decreases in land used for exports, including wheat (−1.96 Mha/yr), sunflower seeds (−0.21 Mha/yr), and sorghum (−0.19 Mha/yr).

In addition to expanding production to meet distant crop demand, capital investments were required to build infrastructure for processing and transporting the increased crop volume. Between 1990 and 2015, Argentina’s telecoupled capital investments shifted from five foreign investors contributing $0.1 million USD per year to nine foreign investors contributing $107 million USD per year.^54^ Between 1990 and 2015, telecoupled capital investments totaled $1.2 billion USD, with the largest investments focused on agricultural development (54%), agro-industry (14%), and agricultural land resources (10%). Overall, telecoupled investments showed a significant positive correlation with land used for crop exports (*p* < 0.05). For every $1 billion USD in telecoupled capital investments, there was an associated 17.0 Mha/yr increase. Capital investments in agro-industry and agricultural development had significant correlations with $1 billion of capital investments, resulting in estimated increases of 88.2 and 25.9 Mha/yr, respectively. Not all investments were associated with increased land use for telecoupled crop exports; capital investments in agricultural water resources showed a significant negative correlation.

The literature review identified additional telecoupled capital investments. Between 1992 and 2012, China’s trade and capital investments with Argentina increased from $160 million USD to $17 billion USD.^55^ This investment expansion (assuming a linear trend over time) in capital investments from China to Argentina showed a significant positive correlation (*p* < 0.05) not only with soybean exports to China, but also with the total land used for crop flows to all of Argentina’s distant trading partners. In 2010, the Argentine government acquired a $10 billion USD loan from a Chinese bank to purchase more trains for its rail network.^55^^,^^56^ Further capital investments from Chinese banks also funded the expansion and maintenance of the national cargo rail systems, with individual investments ranging from $46 million USD to $2.1 billion USD.^57^ Capital investments in expanding cargo rail improved the ability to transport crops from agricultural regions to key shipping ports, an essential component of meeting increased telecoupled demand for distant crops. During the same period (1992–2012), Argentina shipped an additional 5.3 Mtonnes/year of soybeans to China, representing the largest increase in land used for telecoupled crop exports. Capital investments in the infrastructure and processing facilities required for expanded agricultural production supported an increase in land use, while investments in agricultural inputs and agrarian reform helped reduce agricultural land use.

In contrast to the indirect LUC effects of infrastructure-related capital investments, land grabs (capital investments in land purchases) also played a role.^58^ Land grabbing by domestic and international companies has repeatedly been reported in Argentina. However, no centralized database exists regarding foreign land investments and ownership. Despite the lack of centralized data, capital investments have been identified in the literature based on news media, business reports, and governmental reports. In Argentina, Indian companies have acquired over 37,000 ha of olive, peanut, edible oil, and pulse farms since 2000.^59^ None of the farms’ products were exported to India at the investment onset; however, between 2006 and 2008, these products began to be exported to India, with an average of 0.63 Mha/yr used for these crop exports. Within Argentina, other telecoupled capital investments were made by foreign companies from Qatar, Saudi Arabia, and South Korea.^60^ Overall, telecoupled capital investments in Argentina’s agricultural sector led to the development of new agricultural infrastructure, including grain terminals, port docks, and processing facilities, which were crucial for supporting agricultural expansion in the export market.^61^

Increased capital investment did not correspond with an influx of distant migrants to support funded infrastructure projects. Telecoupled migration into Argentina decreased by 288,143 people per year between 1990 and 2015. The largest number of telecoupled migrants between 1990 and 2015 (cumulative) came from Italy (1.3 million), Spain (845,240), and Peru (569,458). Telecoupled migration rates were not correlated with the rate of land used for telecoupled exports or with capital investments.

### Telecoupled impacts on land-use change in India

In 2015, India used 2.56 Mha/yr of cropland for telecoupled exports, sending 75 crops to 169 distant countries. This represented an increase of 1.8 Mha/yr, 30 crops and 77 countries compared to 1990. Land used for telecoupled crop flows showed a significant (*p* < 0.05) positive increase of 0.13 Mha/yr over time. In total, changes in land used for telecoupled exports accounted for 10.7% of the increase in land used for crop flows between 1990 and 2015, and 1.4% of the total harvested crop area in 2015. In contrast to Argentina, India’s land used for telecoupled crop exports was more evenly distributed across crops and receiving countries, suggesting that changes in crop demand from individual countries were less impactful on India’s LUC than global dietary shifts, due to the greater crop diversity. In 2015, the top five receiving countries accounted for 38% (0.98 Mha/yr) of land used for telecoupled exports, with the top five crops accounting for 62% (1.6 Mha/yr) of that land.

Changes in India’s telecoupled crop flows have likely been influenced by shifts in global agricultural markets, altering the competitive landscape. As distant countries changed their agricultural systems and policies, they also altered the competitive market landscape, which, in turn, impacted India’s crop exports. In 2011, India had a comparative advantage for rice, maize, grapes, bananas, onions, and mangoes, which contributed to an increase in land area of 0.03%, 2.95%, 3.58%, 6.19%, 8.79%, and 9.56%, respectively, from 2003 to 2013.^62^ Distant demand and changing competitive markets both contributed to changes in land used for telecoupled exports.

Telecoupled capital investments^54^ originated from 21 donors and totaled $595 million USD per year in 2015, representing an increase of $588 million USD per year and 11 donors compared to 1990. Between 1990 and 2015, telecoupled capital investments totaled $6.6 billion USD, with the largest investments focused on agricultural water resources (33%), agricultural land resources (17%), and agricultural development (16%). Overall, telecoupled capital investments in agriculture showed a significant positive correlation with land used for telecoupled crop exports (*p* < 0.05), resulting in an increase of 4.7 Mha/yr per $1 billion USD. Capital investments in agricultural water resources, agricultural land resources, and agricultural development had significant correlations with $1 billion USD of capital investments, resulting in an estimated increase of 13.8, 24.5, and 15.9 Mha/yr, respectively.

In 2015, 152,524 individuals migrated to India from distant countries, which was a decrease of 5,573 people per year compared to 1990.^63^ The largest flows of migrants were from Brunei Darussalam, Afghanistan, and the United Arab Emirates. The linear regression analysis indicated that telecoupled migration did not have a significant impact on land used for telecoupled exports (*p* > 0.05), and no concurrent changes in crop production were observed between 2000 and 2010 when telecoupled migration decreased. Overall, the land used for India’s telecoupled exports increased significantly from 1990 to 2015 and was positively correlated with telecoupled capital investments.

### Pericoupled drivers of land-use change

The unique dynamics of geopolitical relationships between adjacent countries can impact the flows of crops, capital investments, and migration differently than telecoupled or intracoupled systems. This can result in distinct impacts on land used for pericoupled exports (Table 1). Despite this, significantly less research has focused on pericoupled interactions and their effects on LUC.

In pericoupled systems, crop flows can be impacted by changing trade relationships with adjacent countries, which are driven by regional trade agreements. Regional trade agreements can mitigate the political friction associated with cross-border trade, facilitating increased flows of goods, capital, and people. Regional trade agreements can also increase food access across participating countries by influencing agricultural land-use policies as import and export flows change.^64^ After trade agreements are implemented, flows of crops and other goods increase between adjacent countries, and this affects the land and resources required to produce and transport those goods. In crop trade, trade flows may increase concurrently with agricultural land area, leading to decreases in other land-use categories. On average, yearly deforestation rates have been shown to increase by 19%–26% three years after a trade agreement was enacted, due to the increased conversion of land to agricultural use.^65^ Furthermore, pericoupled crop flows have been shown to increase when restrictions are placed on distant trade.^24^

Capital investments are another flow impacting agricultural LUC in pericoupled systems. Capital investments often flow from higher-income countries to adjacent lower-income countries. For example, China invests heavily in lower-income adjacent countries, where China can apply its agricultural technology to increase yields.^61^ Corporate investment can originate from neighboring or distant countries; however, investments in adjacent countries are often prioritized by investors, as they are perceived as lower risk due to proximity.^66^^,^^67^ Local investors also have a better understanding of the political, social, and economic climate, which again reduces perceived risk. Agricultural investments are often focused on natural resource areas that cross international borders, meaning that investors who already hold land in border regions may expand their landholdings into adjacent countries. Despite the unique dynamics of pericoupled capital investments, there are few records of these investments, limiting the level of quantitative analysis that can be conducted.

Migration, which is often higher from adjacent than distant countries, is another potential cause of agricultural LUC as it impacts both the total population and labor force of a nation. Increased labor force availability can either affect the expansion of agricultural lands or the intensity of agricultural production. Alternatively, a larger labor force can improve labor-intensive crop yields, reducing the land required to maintain production. Since agricultural labor requirements are seasonal, temporary employees migrate during key planting, harvesting, and processing points of the season.^44^^,^^45^^,^^46^ Due to the short-term nature of this work, the migrant workforce is predominantly from lower-wage adjacent countries and bordering agricultural regions, with this labor being an essential part of agricultural production.^68^ One example is in the US, where 68% of farmworkers were from the neighboring country of Mexico.^69^ The dynamics of pericoupled socioeconomic crop flows can be distinct (compared to intracoupled and telecoupled), resulting in unique impacts on LUC.

### Pericoupled impacts on land-use change in Argentina

In 2015, Argentina used 1.8 Mha/yr of cropland for pericoupled exports, sending 58 crops to all five adjacent countries. This represented an increase of 0.6 Mha/yr and 19 crops compared to 1990. Increases in land used for pericoupled exports were not statistically significant (*p* < 0.05) over time between 1990 and 2015. Changes in land used for pericoupled exports accounted for 7.1% of the increases in land used for crop flows between 1990 and 2015 and 5.1% of the harvested crop land area in 2015. Pericoupled flows impacting agricultural LUC were largely impacted by policies related to domestic production and telecoupled trade. Argentina is adjacent to five countries and is a member of two regional trade agreements: the Association for Latin American Integration (Asociación Latinoamericana de Integración, ALADI) and the Southern Common Market (MERCOSUR).^70^

**Figure 3:**
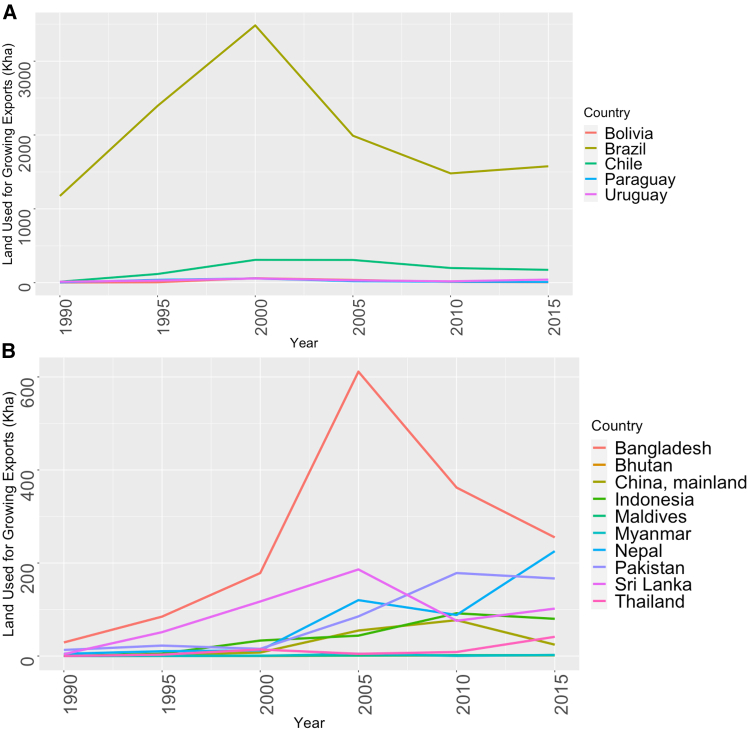
Pericoupled land use Land used for pericoupled crop flows (adjacent country crop flows) from 1990 to 2015 (A) Land used by Argentina to grow crops exported to adjacent countries. (B) Land used by India to grow crops exported to adjacent countries. Data used to create the figure can be found in Table S2.

Regional politics and trade patterns impacted crop flows in Argentina. Between 1990 and 2008, annual soybean production increased by 37 Mt/yr while the annual sowing land area increased by 14.1 Mha/yr.^71^ Pericouplings among Argentina, Paraguay, and Uruguay were substantially altered by China’s increased demand for soybeans and Brazil’s soybean production.^24^ One example was Argentina’s intermediary role in the soybean market in the 2000s, during which the country received a substantial portion of soybean exports from Paraguay and Bolivia. The soybeans were then processed in Argentina and re-exported to distant countries.^72^ After Argentinian export tariffs on soybeans went into effect in 2005, the rapidly increasing exports to China slowed down and were shifted to the neighboring countries of Brazil, Paraguay, and Uruguay (Figure 3). These three countries saw increases in imports from Argentina of 128,766%, 128%, and 88%, respectively, while the corn area planted in Argentina increased by 175%.^24^ Despite the increase in planted area, the land used for pericoupled corn exports actually decreased, indicating that much of the land area increase was used for either telecoupled or intracoupled corn crop flows. In 2015, the majority of Argentina’s pericoupled crop flows went to Paraguay, accounting for 90.4% of land used for pericoupled exports (Figure 3).

Pericoupled capital investments also contributed to the amount of agricultural production and land use in the Chaco and Chiquitano regions, which span Argentina, Bolivia, and Paraguay. In these regions, roughly half of the land investments were from foreign corporations.^73^ Corporate investment in the Argentinian and Bolivian portions of these regions accounted for 2.5 Mha of land use; however, stricter Argentinian deforestation regulations caused a diversion of 7.7% of land investment (0.17 Mha) to the neighboring countries of Bolivia and Paraguay, reducing potential impacts on LUC in Argentina.^73^ Corporate investments in the Chaco and Chiquitano regions were concentrated in areas with fewer deforestation regulations. This meant that if neighboring countries increased their deforestation regulations, there would likely be a corresponding increase in capital investments in land in Argentina, and vice versa.^73^ Pericoupled capital investment flows can move in both directions. Argentina invested in neighboring countries’ agricultural sectors, which potentially lessened the burden on its own natural resources. In Paraguay, Argentina invested in soybeans, wheat, corn, and pasture, contributing to 64% of the soybean production area in the Alto Parana, Canindeyú, Caaguazú, and Itapu departments, which were controlled by foreign countries.^74^ Argentinian companies were the second most common buyers of land in Alto Parana and Itapu departments, with the latter being directly on the Argentinian border.^74^ Argentinian agribusiness companies also invested in soybean and cattle production in Bolivia, Paraguay, and Brazil.^72^

**Figure 4:**
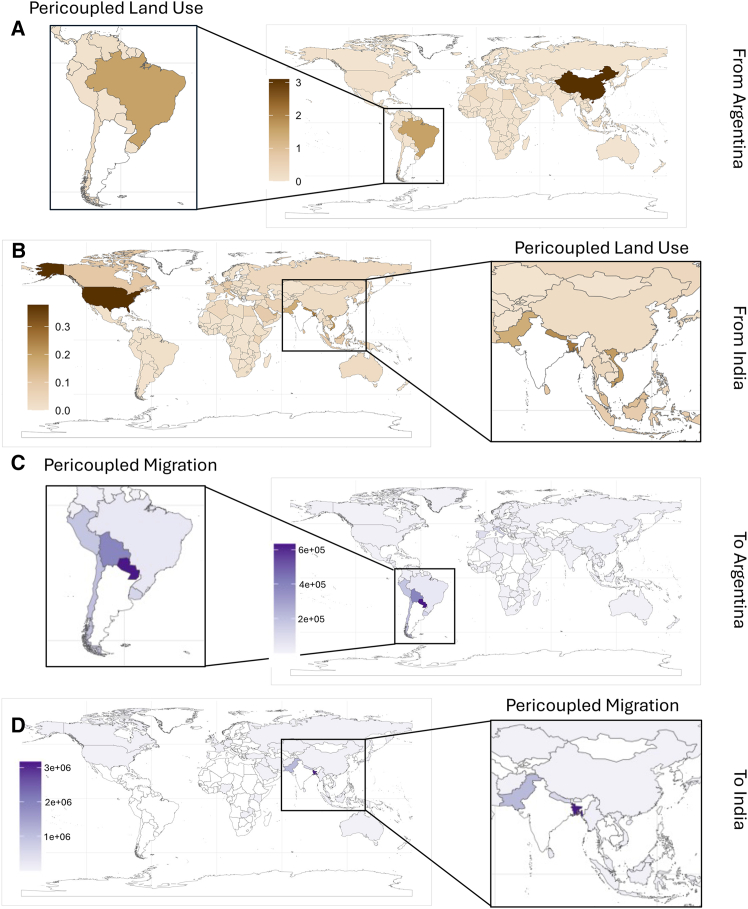
The flows of migration and land use for crop exports for Argentina and India (A) The amount of land (Mha) used for growing crops from Argentina which were exported to each receiving nation. (B) The amount of land (Mha) used for growing crops from India which were exported to each receiving nation. (C) The number of migrants that moved to Argentina in 2015. (D) The number of migrants who moved to India in 2015. Pericoupled land use data were calculated from 2015 FAOSTAT Production and Detailed trade matrix data.^76^^,^^77^ Migration data were for 2015 from the United Nations, Department of Economic and Social Affairs.^63^ Data used to create the figure can be found in Table S3.

Migrant labor from adjacent countries can be another important pericoupling, but was not impactful for Argentina’s agricultural land used for pericoupled exports. Over the past century, Argentina has seen a shift in the origin of migrant populations. Historically, migrants predominantly originated from distant countries, with only 9% coming from neighboring countries in 1914. By 2010, this had shifted to 69% of migrants coming from neighboring countries.^75^ In 2015, 1.2 million people migrated to Argentina from neighboring countries, an increase of 0.3 million people per year compared to 1990.^63^ In 2015, 70.0% of migrants came from neighboring countries, with the top countries being Paraguay, Bolivia, and Chile^63^ (Figure 4). Despite the increase in the percentage of migrants coming from adjacent countries, migration was not significantly correlated with land used for pericoupled exports or overall land used for crop flows. Since Argentina’s LUC was driven predominantly by industrial cereal crops, labor was likely less significant for supporting expansion than it would be in India, which has a larger specialty crop market. Overall, land used for pericoupled exports was driven by regional policies and dynamics, altering the flows of capital investments and crops.

### Pericoupled impacts on land-use change in India

Between 1990 and 2015, India’s land used for pericoupled exports peaked in 2013 at 2.14 Mha/yr, exporting 74 crops to ten countries. This represented an increase of 2.09 Mha/yr, 17 crops, and two countries. Between 1990 and 2015, the amount of land used for pericoupled exports increased significantly (*p* < 0.05) by 0.07 Mha/yr. Overall, changes in land used for pericoupled exports accounted for 5.1% of the increases in land used for crop flows between 1990 and 2015 and 0.5% of total harvested cropland area in 2015. In 2015, 71.8% (0.65 Mha/yr) of land used for pericoupled crop flows were for flows to Bangladesh, Nepal, and Pakistan (Figure 3). Between 1990 and 2015, there was a 0.85 Mha/yr increase in land used for pericoupled crop exports, with one-third of that land used to grow maize and wheat.

India had several key pericoupled flows that contributed to the amount of land used for pericoupled exports. Similar to Argentina, several trade partnerships were active between 1990 and 2015, influencing the flow of crops from India to adjacent countries. In 1992, India, China, and ASEAN (Association of Southeast Asian Nations) Free Trade Area (AFTA) countries (Brunei, Indonesia, Malaysia, the Philippines, Singapore, and Thailand) began expanding their trade partnerships.^78^ Between 2003 and 2005, an average of 44% of India’s imports and 18% of its exports were sent to AFTA or China.^78^ India also increased imports from AFTA by ∼100% ($1 billion USD to $2 billion USD), while AFTA countries doubled their imports from India ($500 million USD to $1 billion USD).^78^ Kallummal and Ratna, 2013, assessed that regional trade agreements had implications on several key crops in India, including pepper, tea, coffee, coconut, and natural rubber.^79^ Regional trade partnerships also impacted India’s trade flows with China. From 2000 to 2006, China’s agricultural imports from India increased by 1,000% ($100 million USD to $1 billion USD), while India’s imports from China increased by only 50% ($200 million USD to $300 million USD).^78^

Pericoupled capital investment data related to agriculture was not available for India. The Land Matrix, which tracks foreign land investments, identified six land investments in India between 1990 and 2015, with only two from adjacent countries, but these were not related to agriculture.^80^ Development flows to Agriculture data,^54^ and the literature review results only include telecoupled capital investments or foreign investments with no specified sending nation. Since capital investments typically flow from higher-income to lower-income countries, China would be the most likely investor in India’s agricultural systems. However, long-standing geopolitical tensions between the two countries may potentially limit those capital investment flows.^81^

Similar to Argentina, pericoupled migration in India was substantially higher than telecoupled migration. In 2015, 4.7 million individuals migrated to India from adjacent countries, accounting for 96.9% of all international migration in 2015. This influx of people can increase domestic food demand (potentially increasing land used for intracoupled crop flows), while an increased supply of labor can help increase yields and potentially decrease land use. Despite the high rates of pericoupled migration between 1990 and 2015, there was a decrease in migration rates of 2.3 million people per year. Pericoupled migration had a significant negative correlation (*p* < 0.05) with changes in land use for pericoupled exports, with decreases in migration correlating to increased land use for pericoupled exports. These shifts demonstrate that changing migration patterns do not necessarily align with changing trade patterns, and that other factors are more likely to impact land used for pericoupled crop flows (i.e., economic growth in receiving countries, a growing export sector in India, etc.). Overall, the combination of changing trade agreements, crop flows, and migration contributed to changes in land used for pericoupled crop exports.

### Intracoupled drivers of land-use change

In addition to flows between countries, flows can also move within a country (intracouplings). Aligning with telecoupling and pericoupling, we evaluated the intracoupled flows of crops, capital investments, and migration. Intracoupled crop flows included any crops that were produced and consumed within a nation. Despite increases in international food trade, the majority of global crops are still produced and consumed domestically.^76^^,^^77^ Domestic and international policies and events can often impact intracoupled crop flows. For example, intracoupled crop flows can increase when there is a spike in food insecurity, as countries often reduce exports and increase domestic production to bolster the domestic food supply.^82^^,^^83^ Other factors that impact intracoupled crop flows can include changes in population, changes in income, conflicts, and natural disasters.^84^

While changes in intracoupled crop flows are most directly linked to the land required for crop production, shifts in migration and capital investment flows can impact production levels and the locations of the land use. Capital investments can occur in intracoupled systems, as domestic businesses and governments invest in infrastructure and irrigation projects, crop insurance and subsidy programs, credit and financing programs, as well as research and extension programs.^85^ These government-based capital investments can have two potential effects on agricultural land use. First, investments focused on expanding agricultural businesses and systems can increase production capacity, leading to the expansion of agricultural industries and land used for crop production. Alternatively, programs focusing on production methods, inputs, and technologies can increase crop yields, reducing the amount of land needed to produce the same quantity of crops.^86^

Domestic migration (the movement of people from one location within a country to another) can lead to shifts in consumption rates and land use patterns. For example, as populations increase and centralize in urban areas, countries convert agricultural land to urban land to meet the growing demand for housing and development. This has been documented in Cairo,^87^ Mexico City,^88^ Osun State, Nigeria,^89^ and Norway.^90^ The combination of converting cropland to urban land and increased populations in urban centers can both redistribute and expand agricultural land to other parts of a country. Intracoupled flows of crops, capital investments, and migration are largely dependent on domestic governmental programs and can have a substantial impact on overall agricultural land use compared to pericoupled and telecoupled systems.

### Intracoupled impacts on land-use change in Argentina

In 2015, Argentina utilized 26.5 Mha/yr of land for intracoupled crop flows, producing 72 crops, which represented a 14.4 Mha/yr increase compared to 1990. Changes in land used for intracoupled crop flows accounted for 85.8% of the increase in total land used for crop flows and 75.2% of total harvested crop land area in 2015. Soybeans and corn had the largest increases in intracoupled land use (12.0 and 1.6 Mha/yr between 1990 and 2015). Intracoupled land use increased significantly over time (*p* < 0.05) with an increase of 0.54 Mha/yr.

Intracoupled crop flows were impacted by domestic governmental policies related to economic goals and international geopolitical factors. In 2006, Argentina implemented an export tax of 23.5% on soybean meal and 19.3% on soybean oil, resulting in slower growth in soybean exports to China.^83^ One of the continued impacts of these taxes was stagnation in land used for telecoupled soybean exports, which had increased by 89% between 2000 and 2006. In contrast, intracoupled land used for soybeans remained relatively constant from 2006 to 2008, then increased by 18% in 2009. Land used for soybeans continued to increase, peaking in 2016 at 16.6 Mha/yr, and accounting for 63% of total land used for intracoupled crop flows.

Changes in crop production levels and flows also depend on capital investments. Between 2001 and 2015, government capital investments peaked in 2008 at $1.5 billion USD per year, with total investments equaling $8.8 billion USD. For increased crop production to reach the market, there must be adequate infrastructure to process and transport crops to potential consumers. In Argentina, increased road development in rural areas created easier opportunities for agricultural land expansion.^91^ These domestic investments were supported by Chinese investments in rail and other transportation infrastructure from 1992 to 2012.^55^^,^^56^ Intracoupled government investments in agriculture were positively correlated (*p* < 0.05) with increases in intracoupled land use between 2001 and 2015, resulting in a 3.8 Mha increase for every $1 billion USD invested.

**Figure 5:**
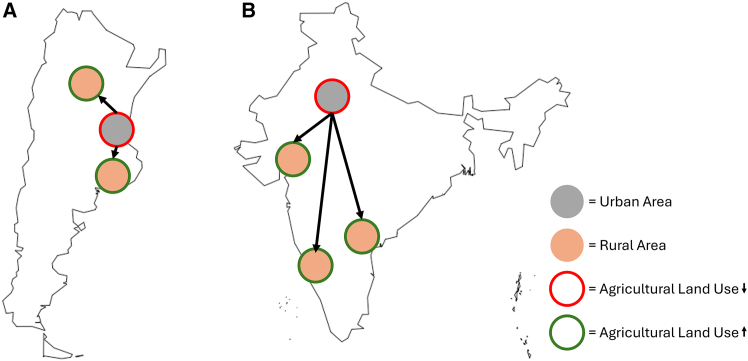
Impacts of intracoupled flows The shift of agricultural land use from around urban areas to rural areas, resulting in the conversion of natural land in rural areas to agricultural land in (A) Argentina and (B) India.

Internal migration flows (rural-to-urban migration) can affect LUC as urban populations grow and urban expansion pushes agricultural land use into new regions of the country. In Argentina, the percentage of urbanization attributed to rural-urban migration decreased from 51% between 1950 and 1960 to 27.6% between 1990 and 2000.^92^ Since data on rural-to-urban migration were unavailable between 1990 and 2015, we report changes in urban populations. Between 1990 and 2015, the urban population of Argentina grew by 11.3 million people, representing a 39.6% increase, and outpaced total population growth. During this time, the rural population decreased by 5.7 million people. Increases in urban population corresponded with increases in urban land use. Between 1985 and 2015, the urban land area around Buenos Aires increased by 93,716 ha, with 30.28% of the area converted from cropland^93^ (Figure 5). Over time, Zalles et al.^94^ observed that cropland conversion shifted further away from urban areas between 2016 and 2018, compared to the period from 1985 to 1994.^94^ As urban land was converted from nearby cropland, the corresponding agricultural LUC occurred in northern forested areas and southern grassland/pasture areas^95^ (Figure 5). Increases in urban population (used as a proxy for rural-urban migration due to data limitations) had a significantly positive correlation with land used for intracoupled crop flows (*p* < 0.05). It is important to recognize that urban population growth and the subsequent expansion of urban land can occur for various reasons and may also be influenced by pericoupled and telecoupled migration rates. Overall, the increase in land used for intracoupled crop flows was supported by government capital investments and increases in urban populations.

### Intracoupled impacts on land-use change in India

In 2015, India utilized 181.2 Mha/yr of land for intracoupled crop flows, producing 79 crops, representing a 14.1 Mha/yr increase from 1990. Intracoupled land use increased significantly over time (*p* < 0.05) with an increase of 0.55 Mha/yr. Changes in land used for intracoupled crop flows accounted for 83.4% of the total increase in land used for crop flows and 98.1% of the harvested crop land area in 2015. Soybeans, wheat, and corn had the largest increases in intracoupled land use (8.8, 7.8, and 3.0 Mha/yr, respectively), between 1990 and 2015. In contrast, sorghum, millet, and ground nuts saw the largest decreases in intracoupled land use (8.3, 6.0, and 3.8 Mha/yr, respectively) between 1990 and 2015.

Investments in technology and infrastructure helped to expand agricultural production across India. Between 2001 and 2015, government capital investments peaked in 2015 at $29.5 billion USD per year into agriculture, forestry, and fishing, with total investments equaling $212 billion USD (data were not disaggregated for agriculture only).^96^ Compared with changes in intracoupled land used for crop production, there was a significant positive correlation (*p* < 0.05) with an increase of 0.74 Mha of land per $1 billion USD invested. These investments helped support innovations in agricultural technologies impacting land use.

As a monsoon country, India experiences prolonged wet and dry seasons, thus making crop production highly dependent on dry-season irrigation. Several innovations in irrigation technology have been introduced to address the lack of rainwater, which has been crucial for agricultural expansion.^97^ Agricultural expansion in western India increased the cropland area by 33.7% from 1982 to 2015, with rates of expansion reaching up to 2% points per year.^98^ During approximately the same time frame (1991–2006), there was a 98% increase in agricultural land area in the Mula Pravara river basin, as previously uncultivated and fallow lands were converted to agricultural use through increased irrigation access.^99^ From 2000 to 2019, cropland expansion occurred predominantly in the northwestern regions of India, which focus on rapeseed production.^100^ Between 1991 and 2000, India (state and central governments) invested 6.0 billion rupees (2004–2005 prices), which increased to 7.7 billion rupees between 2001 and 2010.^101^ Out of those funds, 93.15% were invested in crop production between 2001 and 2010. Private investments also increased, with the gross fixed capital formation as a share of GDP increasing from 5.4% in 1990 to 17.3% in 2010.^102^

Similar to Argentina, India experienced urban land expansion, driven by rural-urban migration, which displaced agricultural land surrounding urban areas. Between 1990 and 2015, the urban population of India increased by 214.3 million people, an increase of 97.0%.^103^ During this period, the rural population increased by 248.7 million. Overall, urban population growth accounted for 40% of total population growth. In 1991, 225.9 million people migrated within the country. Approximately 17.67% (39.9 million) of internal migrants moved from rural to urban areas, which decreased to 16.71% (51.7 million) of total intracoupled migration in 2001.^104^ In 2007, 28.5% (326 million people) of the population migrated within the country.^105^ During this period, the urban land area increased by 73.9% (7.3 Mha).^106^ Research has shown a strong negative correlation between built-up land and cultivated land in India.^99^ One study found that from 1992 to 2012, urban land expansion occurred at a rate of 2.4% per year, with one-quarter of the land transitioning from cropland.^107^ Other studies show that urbanization has occurred primarily on agricultural lands, with seven times more agricultural land than forest land being converted to urban areas between 1880 and 2010.^108^ Large investments in the urban construction sector led to the conversion of natural land to urban land. In contrast, land productivity was the primary driver of conversions from agricultural to urban land.^99^ These results demonstrate how rural-urban migration and the resulting increases in urban LUC have an outsized impact on agricultural lands, displacing them to other regions of the country.

The development of infrastructure to support urban areas can also cause LUC and is often impacted by local policies. Between 1977 and 2014, in Delhi, there was a 30.61% increase in built areas and a 2.41% increase in road and rail networks, while cultivated areas and dense forests decreased by 22.75% and 5.31%, respectively.^109^ Due to this type of expansion, all the agricultural land around Delhi was depleted, meaning that any future increases in crop demand for the region had to be achieved through increased yields or sourced from other areas^110^ (Figure 5). In other regions of India, such as Hyderabad in central India, the amount of agricultural land around urban areas was increasing to meet the food demands of growing population centers.^111^ India’s crop yields increased in the latter half of the 20^th^ century but peaked around 2000.^112^ This suggests that future increases in crop demand will likely need to come from expansion rather than intensification, potentially leading to increased agricultural LUC.

### Combined effects of metacoupled flows

**Figure 6:**
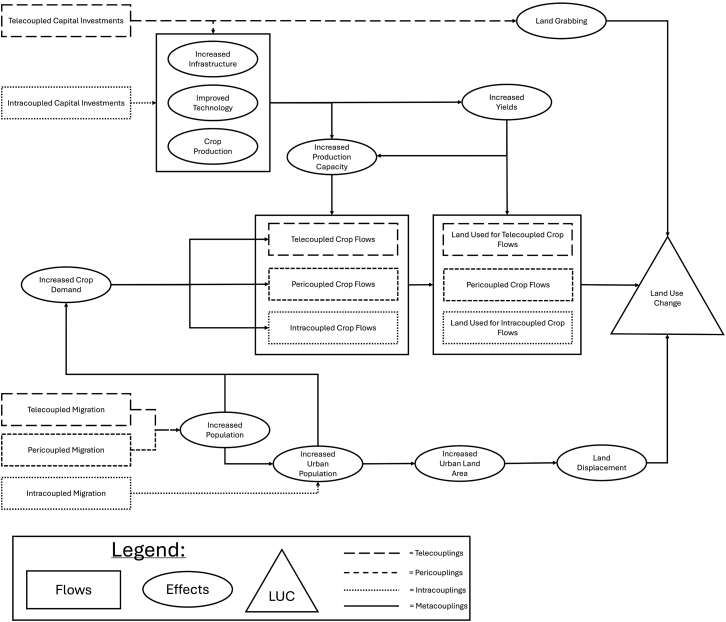
Metacoupled effects of socioeconomic flows The generalized metacoupled effects of socioeconomic flows on the processes impacting LUC. The shape indicates the component of the process with flows (the movement of crops, land used for crop flows, capital investments, and migration) represented by boxes, the effects represented by ovals, and the outcome of LUC represented by the triangle. The dashed lines indicate the coupling, with a solid line indicating that multiple couplings were involved.

Simultaneously assessing how cross-scale couplings (tele-, peri-, and intra-couplings) or metacoupled flows are essential to understanding the dynamics of LUC. In Argentina, the compounding effects of intracoupled and pericoupled migration, as well as telecoupled capital investments, led to an increase in land used for telecoupled and intracoupled crop flows (Figure 6). The telecoupled flows of corn and soybeans in distant countries increased between 1990 and 2015 and were correlated with increases in land used for both telecoupled and intracoupled crop flows. Distant capital investments from China, aimed at increasing transportation infrastructure nationwide, combined with domestic investments in processing facilities, contributed to increases in land used for both intracoupled and telecoupled crop flows. Migration increased across intra- and pericoupled systems, contributing to urban population growth and to the displacement of agricultural land into natural areas north and south of Buenos Aires, Argentina. Overall, between 1990 and 2015, metacoupled crop flows in Argentina (intra-, peri-, and telecoupled) contributed to a 16.8 Mha/yr increase in land used for crop flows and were impacted by telecoupled capital investments, metacoupled migration, and changes in crop demand.

India experienced similar metacoupled dynamics, including intracoupled migration, tele- and intracoupled capital investments, and changing global markets, which affected land used for crop flows. As in Argentina, rural-urban migration contributed to urban population growth and urban land displacement, thereby increasing agricultural land use in other regions of the country. These effects were compounded by tele- and intracoupled investments, which were both positively correlated with the expansion of land used for crop flows. This LUC was exacerbated by the increased land used for crop flows from distant and adjacent countries. Pericoupled migration (despite a decrease in rate between 1990 and 2015) also affected the total population and potentially bolstered the agricultural labor force. Overall, increased metacoupled capital investments and migration helped India expand its agricultural production. This, combined with urban population growth, led to urban land expansion and the displacement of agricultural land, thereby shifting land use from natural to agricultural. Overall, between 1990 and 2015, metacoupled crop flows in India (intra-, peri, and tele-coupled) contributed to a 16.8 Mha/yr increase in land used for crop flows. The combined effects of metacoupled flows in both Argentina and India demonstrate how the impacts of cross-scale socioeconomic flows compound to increase agricultural LUC.

## Discussion

The results of our study highlight the utility of the metacoupling framework for understanding how cross-scale socioeconomic flows affected LUC in a focal country, using case studies of Argentina and India. Despite differences between the two focal countries, both experienced similar patterns in the impacts of socioeconomic flows on agricultural land use. The majority of land used for crop flows was for intracouplings, followed by telecouplings. However, due to Argentina’s specialization in grain production (a crop that is easily transported across larger distances), the ratio of telecoupled to intracoupled crop flows was higher than for India, which produces a higher amount of specialty crops.^76^ India’s higher proportion of land used for intracoupled crop flows likely resulted from its food supply requirements stemming from a larger population, increased agricultural production across the country, and protectionist policies that have prioritized local production and consumption since the 1960 food crisis.^113^

Our results indicate a similar trend in capital investments, with the majority of flows originating from intracoupled sources. Capital investments influence land used for crop production across couplings. For example, the telecoupled capital investments into Argentina’s railway network were combined with intracoupled capital investments into processing facilities. The combination of capital investments in these two components of the crop supply chain was crucial in increasing corn and soybean production capacity, which rose across both telecoupled and intracoupled systems. The interconnection of couplings across scales underscores the importance of evaluating cross-scale flows simultaneously to identify the varying causes and effects that can impact agricultural land use.

By identifying the impacts of cross-scale socioeconomic flows and the governmental policies that can influence them, our research also provides the basis for establishing multipronged policy proposals to address LUC-related challenges. Our results underscore the importance of considering multiple socioeconomic flows across various scales to achieve LUC-related priorities. Several common national and international polices have historically been implemented to address the flows analyzed in this paper. Here, we will outline how, based on our results, these policies may impact LUC and the potential cross-scale interactions. Export tariffs are a common policy used to alter crop flows, aiming to bolster domestic agricultural industries. These policies have been shown to shift trade flows; however, the land used for those crops is likely to be reallocated to other countries or to other crops. For example, the soybean export tariff Argentina implemented in 2005 reduced soybean exports to China, but these flows were redirected to neighboring countries and domestic use, resulting in an overall increase in corn production.^24^ Thus, export tariffs would need to be paired with more focused land-use-related policies to ensure land use is not redistributed and the desired goals are achieved.^114^ Policies surrounding foreign capital investments are becoming increasingly important as countries highly dependent on agricultural imports for food and energy needs have been increasing their capital investments in foreign countries’ agricultural production systems.^115^ Several publications express concerns about the impacts of capital investments, especially in low-income countries, where restrictions are currently centered on domestic laws.^115^^,^^116^ Based on our results, limiting telecoupled capital investments would reduce a nation’s production capacity and decrease the total land used for telecoupled exports. However, it is important to recognize that tele- and intracoupled land use and capital investments are closely intertwined, with telecoupled capital investments enhancing overall production capacity and potentially benefiting national food security or economies. Thus, limiting telecoupled capital investments could negatively affect national development goals related to the agricultural industry. Alternative policy measures could focus on increasing telecoupled capital investments in agricultural technological development, potentially reducing land use by increasing yields.

Migration flows across all couplings contribute to urban population growth and the displacement of agricultural land, leading to the conversion of natural areas. Research studies have emphasized the potential benefits of policies that balance urban expansion with farmland conservation to meet food security goals and have noted that direct land-use controls were more effective in areas facing high urban expansion pressure.^116^ Our results support the creation of comprehensive land-use strategies for countries that balance the priorities of urban expansion, food security, and conservation by identifying and preserving high-productivity agricultural areas to limit the displacement of natural areas.

Our metacoupled analysis also provides insights that can help inform future LUC models. Current LUC models typically use single-scale drivers to predict LUC and are further informed and validated with remote sensing to assess where LUC has occurred.^117^ Both strategies are limited as they do not identify global cross-scale interactions (tele-, peri-, and intracouplings) that can drive LUC. When global models do apply social and economic flows to LUC, limited data may result in missing key agents and causes, leading to a focus on trade and population growth, which have more robust and consistent data sources.^2^^,^^118^^,^^119^^,^^120^^,^^121^ The case studies presented in this paper demonstrate the metacoupling framework’s ability to combine qualitative (e.g., literature reviews) and quantitative data (e.g., FAOSTAT and the United Nations datasets) to identify cross-scale social and economic factors affecting LUC. In addition to the flows presented here, further research should evaluate how technology,^15^^,^^122^^,^^123^ energy,^17^^,^^124^^,^^125^ and culture^17^ flows impact LUC across scales. By expanding the types of flows and the number of focal countries, future research can reveal the metacoupled network of socioeconomic flows, providing essential insights into how tele-, peri-, and intracouplings affect LUC in countries across the globe.

Throughout this paper, we methodically applied the metacoupling framework to assess key cross-scale socioeconomic flows that are impacting LUC. Our results demonstrate how the land used for crop flows and the effects of capital investment and migration flows differ across spatial scales. We demonstrate that analyzing cross-scale socioeconomic flows can provide a nuanced scientific understanding of the potential drivers of LUC. Furthermore, by focusing on socioeconomic flows that national policies can influence, our results provide governments with a decision-making tool for creating and implementing policies. By utilizing the metacoupling framework in this manner, researchers can expand scientific understanding of LUC drivers while providing actionable policy recommendations to address LUC challenges.

### Limitations of the study

**Figure 7:**
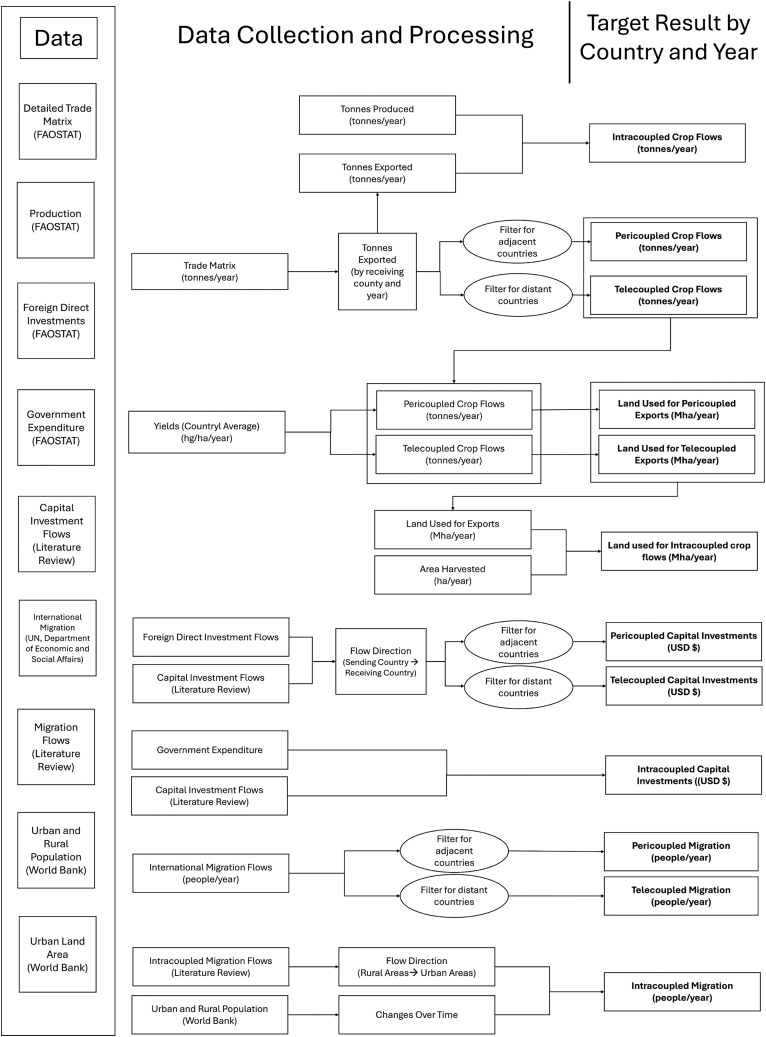
Data collection and processing The data collection and processing methods used to categorize the impact of metacoupled flows on agricultural LUC. All flows were categorized by coupling (tele-, peri-, or intra-coupling), sending and receiving systems, and year.

This paper focuses on the magnitude of crop flows and the compounding impacts of migration and capital investments on land used for crop production. However, due to data limitations, we were only able to identify basic correlations between flows and crop-related land use. We expanded on the quantitative data through a complementary literature review analysis to help explain possible causes for changes in flows and the effects of flows on LUC (Figure 7). Through this method, we were able to identify flows that were not captured in larger datasets and to provide context and logical reasoning for the causes and effects, relating each socioeconomic flow to LUC. However, due to data limitations, we were only able to make correlation-based connections. We were unable to complete a robust analysis that would allow us to identify causal models of how cross-scale flows affect LUC. Additionally, much of the available capital investments data did not specify the sending systems, further limiting our ability to identify the impacts of flows coming from distant or adjacent countries. Due to the varying geopolitical relationships that exist between these two categories of countries, they could have substantially different effects on LUC.

In contrast to other LUC studies, which use remote sensing to quantify LUC, we used a flow-based approach to attribute the embodied impacts of trade on LUC. This methodology has been used in similar applications^39^^,^^41^^,^^126^^,^^127^ and allowed us to attribute the land use embodied in specific crop flows to individual receiving countries. However, it was unable to capture the specific locations where LUC occurred. This method has been utilized in prior research and faced similar data limitations, as crop flow data do not include production locations at a granularity higher than national scales.^41^ Flow-based methods have enabled researchers to attribute the embodied environmental impacts of trade to receiving countries. Still, without higher granularity, they will not be able to link consumption to specific LUC within the sending system directly. Due to the lack of granularity, we were also unable to quantify the land area utilized for sequential multiple cropping systems, which may produce multiple crop harvests within a single year. Our methods assumed that all crops were grown on distinct parcels of land, which double-counted land used for sequential cropping systems. This resulted in a potential overestimation of the total land used for crop flows in India, where two or three harvests can occur on a single parcel in a given year.^128^ However, intercropping systems were accounted for, as yields and trade flows reflected the total area allocated to a specific crop in a given year. Our methods prioritize the accurate quantification of land attributed to crop flows. Our methods were also unable to account for the heterogeneity in yields and the environmental impacts of consumption by product origin. Due to these limitations, we were able to quantify only the land used for crop production at the national scale. We attempted to overcome this limitation by using literature reviews to identify areas that had undergone LUC at the subnational scale.

To overcome the aforementioned limitations and expand the impact of the metacoupling framework, it will be essential to have granular data on flows between distant and adjacent systems, as well as within systems. Granularity of data across all countries is also crucial for the successful application of these research methods. Lower-income countries tend to have less frequent and less granular data collection than wealthier countries. This can significantly limit scientific understanding of the dynamics and impacts of socioeconomic flows across various scales. To maximize the potential of the metacoupling framework for identifying drivers of LUC, future research should simultaneously address current data gaps and incorporate cross-scale flows into LUC models.

## Resource availability

### Lead contact

Any questions or requests for further information should be directed to the lead contact and will be fulfilled by, Joris Van Zeghbroeck (vanjoris@msu.edu).

### Materials availability

This study did not generate new unique reagents.

### Data and code availability

• All data used for the analysis in this article were from publicly available data sources listed in Table S1. Data reported in this article are available from the lead contact upon request.
• The code used to conduct the analyses is available from the lead contact upon request. No other original code was generated as part of this research.
• Any additional items needed to reanalyze the data reported in this study are available from the lead contact upon request.

## Acknowledgments

We would like to thank all of the researchers and staff at the Center for Systems Integration and Sustainability at Michigan State University for their support and input on this work.

Funding was provided by 10.13039/100005825USDA NIFA (2023-68012-39076), 10.13039/100000001National Science Foundation (2118329), AgBioResearch.

## Author contributions

Conceptualization, J.V.Z.; methodology, J.V.Z. and J.L.; investigation, J.V.Z.; writing – original draft, J.V.Z. writing – review and editing, J.V.Z., M.R., N.M., J.L., and E.F.M.; funding acquisition, J.L.; supervision, J.L. and E.F.M.

## Declaration of interests

The authors declare no competing interests

## STAR★Methods

### Key resources table

**Table:** 

REAGENT or RESOURCE	SOURCE	IDENTIFIER
**Deposited data**		

The amount of crops (tonnes/year) sent from a sending to a receiving country.	FAO Detailed trade matrix	https://www.fao.org/faostat/en/#data/TM
The yields (hg/ha) and harvested area (ha) for crops by country and year.	FAO Crops and livestock products	https://www.fao.org/faostat/en/#data/QCL
Disbursements of investments (USD) from a sending to receiving country by year.	FAO Development Flows to Agriculture	https://www.fao.org/faostat/en/#data/EA
The government expenditures (USD) on agriculture, forestry, and fishing.	FAO Government Expenditure	https://www.fao.org/faostat/en/#data/IG
The number of migrants moving from a sending country to receiving country by country and year.	United Nations Population Division and Social Affairs, Population Division	https://www.un.org/development/desa/pd/content/international-migrant-stock
The number of people living in urban or rural areas by country and year	World Bank Group Population estimates and projections	https://databank.worldbank.org/source/population-estimates-and-projections
The amount of urban land area (square km) per year.	World Bank Group Urban land area	https://data.worldbank.org/indicator/AG.LND.TOTL.UR.K2
List of neighboring countries	This Paper	Table S4

**Software and algorithms**		

R	R version 4.0.1	https://www.r-project.org/

### Method details

#### Study area

We applied the metacoupling framework to LUC in two countries, Argentina and India. These countries were selected because of their similarities in ecological importance (e.g., containing global biodiversity hotspots) and economic importance (e.g., leading exporters), and because they are potentially heavily influenced by LUC. Both countries have distinct climates, crop types, and production regions; northwestern Argentina contains most of the cropland and focuses primarily on cereal crops (corn, soybeans, wheat, barley, etc.), while India’s cropland is distributed across the country and has multiple agricultural regions where different crops are grown. By selecting these two countries, this paper provides insights into the drivers of LUC by operationalizing the metacoupling framework.

Argentina’s main agricultural regions are in the Gran Chaco and Chiquitano nature areas, consisting of dry, woodland ecosystems that contain one of the largest remaining continuous areas of native vegetation (700,000 km^2^) in South America. The region has experienced rapid rates of deforestation due to the conversion of forested land to agricultural land, leading to biodiversity loss, soil degradation, and freshwater pollution.^73^^,^^129^ In contrast, India’s agricultural system faces unique challenges due to the rapidly growing population and diverse production regions spread across the country. India’s national land mass pressure (calculated from the ratio of land mass to population) is 4–6 times higher than the global average, as it occupies 2.3% of the global terrestrial area while sustaining 17% of the global population.^130^ Furthermore, India has also seen significant increases in total crop exports, leading to more pressure to continue expanding agricultural land areas. Predictions estimate that cropland expansion and intensification will put ∼30 species at risk of extinction, as well as decrease vegetation and fallow land^131^^,^^132^^,^^133^ land. Both Argentina and India will need to find new ways to meet the rising demands for food and energy, both globally and domestically.

#### Data collection

A mixed methods approach was used to identify agricultural LUC and its drivers by combining existing datasets with a literature review (Figure 7). The amount of land used for crop exports was calculated based on FAOSTAT Trade Matrix data,^77^ which detailed crop flows in tonnes from sending to receiving countries by year, and FAOSTAT Production data,^76^ which contained national-level yield averages and harvested area by year. FAOSTAT data are collected through questionnaires sent to governments and non-governmental organizations and do not include uncertainty values.

Capital investment data were collected from a literature review, development flows to agriculture,^54^ and government expenditure data.^96^ Telecoupled capital investments were obtained from development flows to agriculture^54^ and included sending-receiving system, year (1990–2015), purpose, and amount. We were unable to obtain pericoupled capital investment data as no adjacent countries were included in the development flows to agriculture data, and all other available sources of foreign investments did not specify the sending nation. Government expenditure data^96^ included country, year (2001–2015), and amount (million USD). Additional capital investment data not included in FAOSTAT were obtained from a literature review tracking the value (USD) of capital investments and were coded based on coupling (intra-, peri-, and telecoupled), year, and sending-receiving systems (when applicable).

International migration data from 2015 were obtained from the United Nations’ Department of Economic and Social Affairs,^63^ while domestic migration data were sourced from a literature review and World Bank urban and rural statistics.^103^ We obtained estimates for rural-to-urban migration in India from a literature review; however, we were unable to find matching data for Argentina and instead used urban population changes as a proxy for rural-urban migration.

The literature search was conducted using Google Scholar and the Web of Science Core Collection for the years 1990–2025. The publication year was extended beyond the focus year range to include literature with historical data and results. The literature review included journal publications, gray literature, government and non-governmental reports, and datasets. Quantitative data were extracted and organized into a dataset containing coupling (tele-, peri-, and intracouplings), the sending nation, the receiving nation, the year, the flow, the unit, and the magnitude. The FAOSTAT and UN Population Division and Social Affairs data were summarized into a dataset containing columns matching the literature review data, before being joined by these columns.

All data sources were publicly available, collected with a focus on the years 1990–2015, and analyzed using R version 4.0.1. A complete list of data sources is in the supplemental materials (Table S1), and the code is available upon request.

#### Calculating metacoupled crop flows

##### Calculating land used for crop flows

Data analysis and modeling of agricultural land flow impacts were conducted based on the processes outlined in Figure 7. Crop flows and land use were calculated based on the FAOSTAT trade matrix and production data. The datasets were merged using the UN country codes of the sending country and production nation, the item name, and the year (Figure 7). The land used for crop exports (*Z*) of a crop (*c*) in year (*t*) to a receiving country (*r*) from a sending country (*s*) was calculated based on the export quantity (*E*) and yields (*Y*) as described in Equation 1:

(Equation 1) $$Z_{c,t,r,s}=\frac{E_{c,t,r,s\;}}{Y_{c,t,s}}$$

##### Calculating land used for telecoupled and pericoupled crop flows

The land used for exports was further summarized for all crops from 1990 to 2015 for all receiving countries, adjacent receiving countries (pericoupled) (*a*), and distant receiving countries (telecoupled) (*d*) as described in Equations 2 and 3 (a list of adjacent countries used for the analysis is available as Table S4).

(Equation 2) $$Z_{a}=\sum_{c=1}^{C}\sum_{t=1990}^{2015}\sum_{r\in N\left(a\right)}^{R}\left(E_{c,t,r,s}/Y_{c,t,s}\right)$$

(Equation 3) $$Z_{d}=\sum_{c=1}^{C}\sum_{t=1990}^{2015}\sum_{r\in N\left(d\right)}^{R}\left(E_{c,t,r,s}/Y_{c,t,s}\right)$$

##### Calculating land used for intracoupled crop flows

In addition to land used for crop flows across adjacent and distant systems, we calculated land used for crop production that remained within a country (land used for intracoupled crop flows). Land used for intracoupled crop flows (*Z*_*i*_) was calculated by crop item (*c*) based on total harvest area (*H*) and total land used for exports (*E*) for a given crop for a given year (*t*) (Equation 4).

(Equation 4) $$Z_{i}=\sum_{C=1}^{C}\sum_{t=1990}^{2015}\left(H_{c,t}-E_{c,t}\right)$$

### Quantification and statistical analysis

We modeled agricultural land-use flows over time using a linear regression model (Equation 5). This enabled us to determine whether there was a significant change in land used for crop flows over time and whether there was a significant correlation between land used for crop flows and other socioeconomic flows. Each flow type was classified as the predictor variable, while land used for crop flows was the response variable. We did not incorporate lag models due to the limited data available for each model and because the goal was to identify possible connections rather than establish causality. We used a *p*-value of 0.05 to determine significance.

(Equation 5) $$y=\beta_{0}+\beta_{1}x+\epsilon$$

## Supplemental information

Document S1. Table S1

Table S2. Data used to create Figure 3; Argentina and India pericoupled land use

Table S3. Data used to create Figure 4; map data

Table S4. List of neighboring countries
