# Supplementary material for: Agriculture land-use change is driven by socioeconomic flows across local to global scales
Source: iScience. 2026 Mar 2;29(4):115200. doi: 10.1016/j.isci.2026.115200 (PMC13010114; doi:10.1016/j.isci.2026.115200)
Supplement: Document S1. Table S1 [file mmc1.pdf]

iScience, Volume *29*

Supplemental information

Agriculture land-use change is driven

by socioeconomic flows across local to global scales

Joris Van Zeghbroeck, Michele Remer, Nicholas Manning, Emilio F. Moran, and Jianguo Liu

Table S1. **Data sources.** The data sources used in the research, with descriptions of their use and the source.

Data Sources	Description	Calculation Use	Citation
FAOSTAT Detailed Trade Matrix Data	The amount of crops (tonnes/year) sent from a sending to a receiving country.	Tele-, Peri-, and Intracoupled Crop Flows	FAO Detailed trade matrix. (2025). [Dataset]. https://www.fao.org/faostat/en/#data/TM
FAOSTAT Crops and livestock products	The yields (hg/ha) and harvested area (ha) for crops by country and year.	Tele-, Peri-, and Intracoupled Crop Flows	FAO Crops and livestock products. (2025). [Dataset]. https://www.fao.org/faostat/en/#data/QCL
FAOSTAT Development Flows to Agriculture	Disbursements of investments (USD) from a sending to receiving country by year.	Telecoupled Capital Investments	FAO Development Flows to Agriculture. (2025). [Dataset]. https://www.fao.org/faostat/en/#data/EA
FAOSTAT Government Expenditure	The government expenditures (USD) on agriculture, forestry, and fishing by country and year.	Intracoupled Capital Investments	FAO Government Expenditure. (2025). [Dataset]. https://www.fao.org/faostat/en/#data/IG
United Nations International Migrant Stock	The number of migrants moving from a sending country to receiving country by country and year.	Tele- and Pericoupled Migration	United Nations Population Division and Social Affairs, Population Division. (2024). International Migrant Stock 2024 [Dataset]. https://www.un.org/development/desa/pd/content/international-migrant-stock
World Bank Population Data	The number of people living in urban or rural areas by country and year	Intracoupled Migration	World Bank Group. (2025). Data Bank, Population estimates and projections [Dataset]. https://databank.worldbank.org/source/population-estimates-and-projections
Urban land area (sq. km) Data	The amount of urban land area (square km) per year.	Intracoupled Land Use Change	World Bank Group. (2025). Data Bank, Urban land area (sq. km) [Dataset]. https://data.worldbank.org/indicator/AG.LND.TOTL.UR.K2
